# Preanalytical, Analytical and Postanalytical Analyses on *Corynebacterium* spp. and Actinomycetaceae in Urine Samples of Patients with Suspected Urinary Tract Infection—A Hypothesis-Forming Observational Study

**DOI:** 10.3390/diagnostics14070746

**Published:** 2024-03-30

**Authors:** Hagen Frickmann, Kerstin Schwinge, Andreas Podbielski, Philipp Warnke

**Affiliations:** 1 Institute for Medical Microbiology, Virology and Hygiene, University Medicine Rostock, 18057 Rostock, Germany; kerstinschwinge@yahoo.de (K.S.); a.podbielski@gmx.net (A.P.); 2 Department of Microbiology and Hygiene, Bundeswehr Hospital Hamburg, 20239 Hamburg, Germany

**Keywords:** urinary tract infection, urine sample, Gram-positive rod-shaped bacteria, *Corynebacterium*, *Actinomyces*, *Winkia*, *Actinobaculum*, *Actinotignum*

## Abstract

A hypothesis-forming exploratory cross-sectional assessment was conducted to assess the occurrence and relevance of Gram-positive rod-shaped bacteria like *Corynebacterium* spp. and Actinomycetaceae in human urine samples. In total, 1170 urine samples from 1031 inpatients with suspected urinary tract infection were assessed for culture-based growth of Gram-positive rod-shaped bacteria applying API Coryne assays, matrix-assisted laser desorption–ionization time-of-flight mass spectrometry (MALDI-TOF-MS), and in-house 16S rRNA gene sequencing. Overall, 502 different bacterial colonies from 346 urine samples taken from 324 inpatients were observed. The three quantitatively most abundant genera or genus clusters were *Corynebacterium* (254 isolates, 62%), *Actinomyces*/*Winkia* (79 isolates, 19%), and *Actinotignum*/*Actinobaculum* (29 isolates, 7%). Compared to sequencing, the diagnostic accuracy of all assessed competitor assays from the diagnostic routine was <80% for differentiation on the genus level and <30% for differentiation on the species level. Prolongated incubation for 4 days compared to 2 days resulted in additional detection of 15% of the totally recorded Gram-positive rod-shaped bacteria. An approximately 5-fold increased detection rate in mid-stream urine compared to urine acquired applying alternative sampling strategies was observed. In conclusion, in the rare event of the suspected clinical relevance of such findings, confirmatory testing with invasively sampled urine should be considered due to the high contamination rate observed in mid-stream urine. Confirmatory testing by DNA-sequencing methods should be considered if an exact identification of genus or species is regarded as relevant for the individual choice of the therapeutic strategy.

## 1. Introduction

The distal structures of the human urogenital tract are extensively physiologically colonized by bacteria and to a lesser extend also its proximal elements [1,2]. Accordingly, urine samples are prone to containing these bacteria. In the case of suspected urinary tract infection, this affects the decision on etiological relevance for the detected microorganisms. In addition, typical causative agents of urinary tract infections are characterized by “facultative pathogenicity”. This term describes a microorganism’s ability to act either as a harmless colonizer or as an agent able to initiate etiologically relevant infections depending on environmental and host-defense factors. Thereby, the concept of facultative pathogenicity is in pronounced conflict to the third historic Henle–Koch postulate. The latter said that pure cultures of an infectious agent should be sufficient conditions for the experimental induction of an associated infectious disease [3]. The fulfillment of this postulate would nowadays be called “obligate pathogenicity”. Such “obligate pathogenicity” is virtually never the case for microorganisms causing urinary tract infections, except for some rare exemptions like the mycobacteria in the tuberculosis complex.

However, urinary tuberculosis is infrequent in western industrialized countries. Instead, *Escherichia coli*, followed by enterococci, staphylococci, and Enterobacterales, different from *E. coli* like *Klebsiella* spp. and *Proteus* spp., are most frequently associated with non-nosocomial urinary tract infections [4,5]. In the case of nosocomial infections, the spectrum is slightly different with a broad spectrum of Enterobacterales next to enterococci and staphylococci as well as—to a lesser extend—*Pseudomonas aeruginosa* and *Candida albicans* being considered as potentially relevant [4,5].

However, all these species can be isolated as contaminants as well. To circumvent this problem, complex diagnostic interpretation guidelines like the German MiQ (“Mikrobiologisch-infektiologische Qualitätsstandards”/“Micrological/infectiological quality standards”) documents [5] include various factors like absolute quantification, consideration of pre-analytic features like the sampling strategy, as well as storage and transport conditions, etc., to provide recommendations for the assignment of likely etiological relevance for a diagnostic result.

Previously published works [6,7,8,9,10] strongly discourage microbiological urine assessments in the absence of inflammatory signs like leukocyturia, positive leukocyte esterase reaction or positive nitrite reaction in urine to reduce superfluous antimicrobial therapies in response to the misinterpretation of contaminants. However, the available interpretation criteria are based on probabilistic evidence. The variety of factors influencing the results of microbiological urine diagnostics can nevertheless lead to indistinguishable results both resulting from infection or colonization on an individual scale.

To provide an example of such multiple realizability, a high pathogen load of a pure culture of *E. coli* in urine, which can typically be found in the case of a “standard” urinary tract infection, could also be caused by a single species contamination combined with unfavorable storage and transport conditions. As shown decades ago, such unfavorable storage and transport conditions can alter pathogen loads in biological samples in different directions. This includes an increased die-off of particularly vulnerable microbial species, as well as overgrowth by less vulnerable ones [11].

Assignment of etiological relevance becomes even more challenging in the case of the detection of bacteria in diagnostic urine samples which have been described to infrequently cause urinary tract infections but which are very frequently part of the human resident flora [12]. Gram-positive rod-shaped bacteria are typical examples. Focusing on *Corynebacterium* spp., species like *C. amylocatum*, *C. aurimucosum*, *C. glucoronolyticum*, *C. urealyticum*, and *C. pseudogenitalium*, the latter also contributing to the formation of urinary stones due to high urease activity and associated alkalization of the urine, have been associated with urinary tract infections in previous works [13,14,15,16,17,18,19,20]. Actinomycetaceae have been reported as potentially relevant as well [21,22,23,24,25,26,27,28]. For *Actinomyces* spp., opportunistic infections of the urogenital tract have been associated with injuries to the skin or mucous membrane barrier due to trauma or surgery [21]. For *Winkia neuii* (formerly *Actinomyces neuii*), biofilm-associated urinary tract infections in the case of indwelling foreign materials have been reported [22,23]. For some *Actinotignum* spp. and *Actinobaculum* spp. like *Actinotignum schaalii* (formerly *Actinobaculum schaalii*), severe urinary tract infections with restricted treatment options due to highly resistant strains have been described [24,25,26,27,28]. Keeping the hypothetical etiological relevance even of such rarely diagnosed causes of urinary tract infections in mind, some authors have advocated not to regard mixed flora from urine as contamination, but to identify all encountered species [29,30]. However, such minority opinions need to be carefully balanced against associated risks of overtreatment and antimicrobial resistance selection as stated above [6,7,8,9,10]. This consideration stresses the importance for a rational assessment of the likely etiological relevance or irrelevance of diagnostic results.

To facilitate estimations of their potential etiological relevance, it is useful to have broader information on the expected frequency of isolations of Gram-positive rod-shaped bacteria from diagnostic urine samples. However, respective studies are scarce and diagnostic surveillance data might be compromised due to the fact that Gram-positive rod-shaped bacteria are frequently neglected during the diagnostic workup of urine samples, particularly when occurring as part of mixed bacterial flora [5]. Accordingly, a cross-sectional study was conducted to assess the abundance of Gram-positive rod-shaped bacteria in urine samples of inpatients with suspected urinary tract infections at a German tertiary hospital, with particular focus on *Corynebacterium* spp. and Actinomycetaceae. Some genera like *Bifidobacterium* spp., *Gardnerella* spp. and *Lactobacillus* spp. were deliberately excluded as components of the physiological urethral flora [31,32].

Also, the reliability of commonly applied diagnostic approaches, the effect of incubation time as well as preanalytical influences like the mode of urine sampling or factors with relevance for postanalytical interpretations like patient age and sex were assessed.

## 2. Materials and Methods

### 2.1. Study Design, Study Population and Assessed Preanalytical and Postanalytical Parameters

The study was designed as a hypothesis-forming explorative cross-sectional assessment. The analyses were conducted with urine samples collected within an eight-month study period at a Germany tertiary hospital from inpatients with suspected urinary tract infections. The recorded preanalytical parameters comprised the urine sampling strategy, including mid-stream or first jet urine, urine from an indwelling or an intermittent catheter, urine collected in a pot or a pouch, urine from an entero-vesical fistula or an ileal conduit, or urine invasively sampled via puncture of the bladder or the renal pelvis, as well as situations with insufficient information on the applied sampling approach. The collected patient data used for postanalytical assessments comprised age stratified by decades and sex.

### 2.2. Assessed Analytical Parameters, Inclusion and Exclusion Criteria

All assessed diagnostic urine samples were initially analyzed in a microbiological diagnostic routine laboratory accredited according to DIN EN ISO 15189 [33], which included cultural growth of aerobic bacteria for 40–48 h at 36 (±1) °C on Colombia agar enriched with 5% sheep blood (bioMerieux, Nürtingen, Germany). Instead of being disposed after the routine diagnostic procedures, the agar plates were qualitatively screened visually by experienced investigators for colony morphology of potential Gram-positive rod-shaped bacteria as part of this study. Colony morphologies untypical for Gram-positive rod-shaped bacteria as judged by the investigators were not analyzed further. Afterwards, the agar plates were incubated for additional 40–48 h at 36 (±1) °C, resulting in a second assessment after a total of 80–96 h. Exclusion criteria included the overgrowth of the agar medium by molds, negatively interfering with the detectability of colonies of Gram-positive rod-shaped bacteria. In case of overgrowth by Gram-negative bacteria, e.g., Enterobacterales like *Proteus* spp. showing swarming behavior, isolation on CNA agar (bioMerieux, Nürtingen, Germany) was used to suppress Gram-negative bacterial growth.

Suspected colonies were assessed with the diagnostic approaches as described in the “Analytical workflow” subheading below. Gram staining was conducted using a PREVI Color V2 automatic device (bioMerieux, Nürtingen, Germany) as recommended by a manufacturer.

Matrix-assisted laser desorption–ionization time-of-flight mass spectrometry (MALDI-TOF-MS) analysis was performed using an Axima Assurance device (Shimadzu, Kyoto, Japan). The obtained spectra were analyzed using the database Saramis version 15.10.13–18.11.14 (bioMerieux, Nürtingen, Germany) and the database Myla version 15.09.–24.11.2014 (bioMerieux, Nürtingen, Germany). Biochemical assessment was conducted using the API Coryne assay (bioMerieux, Nürtingen, Germany) according to the manufacturer’s instructions.

As a reference method for this study, an in-house 16S rRNA gene sequencing protocol was applied. In detail, DNA extraction from pure colony material was conducted applying the QIAamp DNA Mini Kit (Qiagen, Hilden, Germany). Afterwards, 16S rRNA gene-based pan-bacterial PCR as described in [34] was run using the forward primer 16S8_27 (5′-AGAGTTTGATCMTGGCTCAG-3′) and the reverse primer 16S519 (5′-GWATTACCGCGGCKGCTG-3′). The run conditions comprised initial denaturation at 95 °C for 3 min followed by 30 cycles at 94 °C, 50 °C and 72 °C for one minute each with a subsequent final extension step at 72 °C for 5 min. Purification of obtained amplicons was based on agarose gel electrophoresis using 1.2% agarose gels. Subsequently, the amplicons were sent for commercial Sanger sequencing to the company Seqlab (Microsynth AG, Göttingen, Germany). Quality-control of the returned sequence files comprised assessment with the software Finch Trace Viewer version 1.4.0 (Geospiza Inc., Seattle, WA, USA). Afterwards, analysis using the Basic Local Alignment Search Tool (BLAST) provided by the National Center for Biotechnology Information (NCBI) [35] was conducted. For the interpretation of the database results, matches ≥ 99% for sequence identity were accepted as identifications at the species level, whereas matches between ≥97% and <99% for sequence identity were accepted as identifications at the genus level. In cases where repetition was required for the diagnostic steps, isolates were deep-frozen at −80 °C in cryotubes (Pro-Lab Diagnostics, Richmond Hill, ON, Canada).

### 2.3. Analytical Workflow

The analytical workflow in case of observation of bacterial colonies suspected of resulting from Gram-positive rod-shaped bacterial growth is summarized in Table 1. In short, Gram-staining was used to exclude Gram-morphologies other than Gram-positive rod-shaped or coccoid bacteria. Afterwards, MALDI-TOF-MS was used to further exclude non-target organisms including species like *Bifidobacterium*, *Gardnerella vaginalis* or *Lactobacillus*, which were considered out of focus for the present assessment. Biochemical results using the API Coryne approach and 16S rRNA gene sequencing, of which the latter was used as reference testing, were added, before Gram-positive rod-shaped bacterial isolates were finally subjected to deep-freeze storage at −80 °C as Microbank-cryostocks (Pro-Lab Diagnostics, Round Rock, TX, USA).

### 2.4. Taxonomical Nomenclature Use

The databases applied for the identification of the bacteria partly provided genus and species names which now have to be considered as outdated. In order not to alter the obtained study results, the outdated nomenclature as provided by the databases is used in the tables in Appendix A of this work. Whenever necessary for understanding the study results, reference to the up-to-date taxonomy is provided in the main manuscript text and its tables. For the presented study, nomenclature changes within the family Actinomycetaceae and, thereby, within the genera *Actinomyces* and *Winkia*, as well as within the genera *Actinotignum* and *Actinobaculum* [36], are of particular relevance. Therefore, *Actinomyces* and *Winkia* as well as *Actinotignum* and *Actinobaculum* are used as genus clusters in the following sections.

### 2.5. Ethics

Ethical clearance for the study was obtained from the ethics committee of the Medical Faculty of the University of Rostock (reference number A2019-0021), which allowed the anonymized data assessment without informed consent. The study was conducted in line with both National German laws and the Declaration of Helsinki and all its amendments.

## 3. Results

### 3.1. Characterization of the Study Population

Within an 8-month study interval, a total of 1170 urine samples from 1031 inpatients with suspected urinary tract infection at a tertiary hospital were included in the assessment, among them follow-up samples from a total of 139 patients. Details on the composition of the study population are indicated in Table 2. In short, there was a quantitatively moderate dominance of male patients. The mean patient age was 54 years in a right-shifted distribution, with the youngest patient being in the first and the oldest in the 96th year of age at the time of the assessment.

### 3.2. Analytical Assessments on Gram-Positive Rod-Shaped Bacteria in Diagnostic Urine Samples

A total of 502 different bacterial colonies from 346 urine samples taken from 324 inpatients were suspected of containing Gram-positive rod-shaped bacterial growth and thus subjected to further downstream analysis. Applying the diagnostic workflow and the exclusion criteria as indicated in the Methods section, the number of isolates subjected to diagnostic MALDI-TOF mass spectrometry, biochemical assessment based on the API Coryne assay, and 16S rRNA gene sequencing were *n* = 441, *n* = 452, and *n* = 429, respectively. The higher number of API Coryne assessments in spite of this assay’s subordinate position in the downstream analysis of the diagnostic workflow is a consequence of the inclusion of results from the routine diagnostic setting, resulting in API Coryne assessments of 320 urine samples taken from 302 patients.

Focusing on the excluded isolates, 61 out of 502 initially selected suspected colonies were a priori excluded on the MALDI-TOF MS detection level due to non-matching Gram staining results or as non-target organisms like *Bifidobacterium* species, *Gardnerella vaginalis*, or *Lactobacillus* species. Based on the Myla database results of the MALDI-TOF assessments, a further 50 isolates were excluded as non-target organisms. From 429 isolates finally subjected to diagnostic 16S rRNA gene sequencing, another 17 isolates were excluded as non-target microorganisms, resulting in a total of 412 sequence-confirmed Gram-positive rod-shaped bacteria, isolated from 298 urine samples taken from 282 inpatients used for post-analytical downstream analysis.

Among the sequence-confirmed Gram-positive rod-shaped bacteria, the three quantitatively most abundant genera were *Corynebacterium* (254 isolates, 62%), *Actinomyces*/*Winkia* (79 isolates, 19%), and *Actinotignum*/*Actinobaculum* (29 isolates, 7%). The numbers of assessed isolates without conclusive results applying MALDI-TOF-MS using the Saramis database, MALDI-TOF-MS using the Myla database, API Coryne-based biochemistry, and 16S rRNA gene sequencing were *n* = 326 (74%), *n* = 89 (20%), *n* = 1 (0%), and *n* = 21 (5%), respectively. Of note, 16S rRNA gene sequencing-based differentiation succeeded for 284 isolates missed by the Saramis database and for 75 isolates missed by the Myla database.

Details on the diagnostic results obtained with the different diagnostic approaches are provided in Appendix A, Table A1. In summary, considerable mismatching of the results of the routine diagnostic standard procedures MALDI-TOF-MS and API-Coryne-based biochemical assessment compared to 16S rRNA gene sequencing, which was used as a reference method for this study, were seen for the assessed Gram-positive rod-shaped bacteria. In addition, the matching of MALDI-TOF-MS results with 16S rRNA sequencing relevantly depended on the used MALDI-TOF-MS database. MALDI-TOF-MS- and biochemistry-based identifications were confirmed by 16S rRNA sequencing in 14.3–29.1% of the cases at the species level and in 51.5–65.2% of the cases on genus level. While the applied Saramis database allowed for better matching of MALDI-TOF-MS results with 16S rRNA sequencing on genus level compared to the Myla database, the opposite was the case for discriminations at the species level (Table 3). Additionally, the Myla database had a lower rate of a priori non-interpretable results as mentioned above.

Focusing on the genera *Corynebacterium*, *Actinomyces*/*Winkia*, and *Actinotignum*/*Actinobaculum*, 16S rRNA sequence-based confirmation of MALDI-TOF-MS-based and API Coryne-based differentiation was accomplished at the species level in 5.9–15.7%, 3.8–29.1%, and 0–6.9% of the cases, respectively, and at the genus level in 33.1–71.3%, 12.7–75.9%, and 0–20.7% of the cases, respectively. More reliable MALDI-TOF-MS differentiation results were obtained with the Myla database for the genera *Corynebacterium* and *Actinomyces*, while the Saramis database was more reliable for the identification of *Actinotignum*/*Actinobaculum*. Details are provided in Table 3.

When focusing on the duration of incubation, prolongated incubation from 48 h to 96 h did not increase the detection rate of Gram-positive rod-shaped bacteria for 85% of the samples. For 15%, however, additional growth of such microorganisms could be confirmed after 96 h of incubation. These 15% comprised 11% of cases in which additional Gram-positive rod-shaped bacteria grew after 96 h, although other colony morphologies of different Gram-positive rod-shaped bacterial species had been observed already after 48 h, while in 4% of the cases growth of Gram-positive rod-shaped bacteria was first detected after 4 days of incubation. Details on the distribution are shown in Appendix A, Table A2; an overview on the bacteria isolated after 96 h incubation is provided in Appendix A, Table A3 and Table A4.

### 3.3. Preanalytical Assessments on Gram-Positive Rod-Shaped Bacteria in Diagnostic Urine Samples

Preanalytical assessments were focused on associations of the urine sampling strategy and the culture-based detection of Gram-positive rod-shaped bacteria. As detailed in Table 4 and Appendix A, Table A5, the vast majority of Gram-positive rod-shaped bacteria were isolated from non-invasively taken urine samples like mid-stream urine, first jet urine, urine collected in a pot, or urine samples acquired by catheterization. In contrast, detecting Gram-positive rod-shaped bacteria were rare events from invasively acquired urine samples, e.g., urine sampled via puncture of the bladder or the renal pelvis. Of note, nearly half of the mid-stream urine samples showed growth of Gram-positive rod-shaped bacteria, while this was the case for only about 10% of all urine samples. Again, *Corynebacterium* spp. and Actinomycetaceae quantitatively dominated.

### 3.4. Postanalytical Assessments on Gram-Positive Rod-Shaped Bacteria in Diagnostic Urine Samples

Postanalytical assessments were focused on associations of detections of Gram-positive rod-shaped bacteria with sex and age of the study population. As detailed in Table 5 and Appendix A, Table A6, *Corynebacterium* spp. were slightly more frequent in urine samples of females, while *Actinomyces*/*Winkia* spp. were more often isolated from male patients. For the Gram-positive rod-shaped bacteria in total, the female:male ratio was 45:55. In more detail, *C. aurimucosum* and *Corynebacterium* “Smarlab Biomol” were more frequently found in samples from female patients, whereas *A. turicensis*, *A. radingae*, *C. glucoronolyticum*, *C. jeikeium*, *C. pseudogenitalium*, and *C. tuberculostaericum* were more frequently found in samples from male patients.

Regarding the associations of Gram-positive rod-shaped bacteria with the age of the study population (stratified by decades), details are provided in Table 6 and Table 7 as well as in Appendix A, Table A7. In short, Gram-positive rod-shaped bacteria were most common in the age ranges of 31–40, 41–50, and 51–60 years (one detection per 2.3 urine cultures) and least frequent in the ranges of 81–90 years (one detection per 4.1 urine cultures). Also, there was a tendency for fewer detections in the first age decade. For *Corynebacterium* spp. and *Actinomyces*/*Winkia* spp., no age association was observed. For *Actinotignum*/*Actinobaculum* spp., in contrast, detections occurred more frequently in patients older than 30 years.

## 4. Discussion

The study was performed as a broad assessment on Gram-positive rod-shaped bacteria in human urine samples, providing a representative data basis by assessing a relevant number of samples with routine diagnostic methods in an accredited laboratory. This approach led to a number of results.

Focusing on the analytical assessments, it could be shown that commonly applied routine diagnostic approaches like MALDI-TOF-MS or semi-automated biochemical assays showed imperfect diagnostic reliability for discrimination at the genus level and even less reliable results at the species level when applied with Gram-positive rod-shaped bacteria isolated from urine samples compared to 16S rRNA gene sequencing. For MALDI-TOF-MS results, the diagnostic accuracy largely depended on the quality of the applied database as confirmed by the comparison of the Saramis and the Myla approach. In our hands, the API Coryne assay still showed good matching with 16S rRNA gene sequencing for individual, potentially relevant species like *C. glucoronlyticum*, *C. urealyticum*, *A. turicensis*, and *A. radingae* (details in Appendix A, Table A1). For other identification results, the matching was considerably worse.

The observed limitations of microbiological diagnostic standard approaches when applied with rarely differentiated Gram-positive rod-shaped microorganisms are well-known from previous studies [37,38,39,40,41,42], and resulting minor and major detection errors are not surprising in this respect. Consequently, surveillance assessments on Gram-positive rod-shaped bacteria based solely upon routine diagnostic results should be interpreted with care and medical microbiologists need to be aware of diagnostic failure in the routine situation when relying on these methods.

However, there are also evidence-supported reasons not to overestimate the reliability of diagnostic 16S rRNA sequence assessment based on publicly accessible databases, as carried out in this study, which are not quality-controlled for in vitro diagnostic use. It has been repeatedly shown that sequences within such public databases have been erroneously assigned [43,44,45,46,47,48], resulting in the risk of non-conclusive or even false diagnostic results as well. Although 16S rRNA gene sequencing has been applied as diagnostic reference approach in the here presented study, the correctness of its results cannot be definitely considered as guaranteed, a residual uncertainty of this work which is methodically immanent. To resolve this problem, quality-controlled diagnostic sequencing solutions labeled for in vitro diagnostic use also covering rarely isolated pathogens would be highly desirable for microbiological routine diagnostic laboratories. This is so far an unmet diagnostic need.

Focusing on the incubation time, it could be shown that the majority of Gram-positive rod-shaped bacteria could be detected after 48 h of incubation, while only a minor effect could be achieved by prolongated incubation for 96 h. Thus, the work is in partial contradiction to previous assessments favoring prolongated incubation of Gram-positive rod-shaped bacteria for about five days [49,50]. It can only be speculated whether growth-supporting matrix effects of the urine samples could have played a role in the here-presented study. Hypothetically, Gram-positive rod-shaped bacteria might remain more vital in the moist environment of urine during sampling and storage compared to other sample materials.

Regarding the preanalytical stratification by the mode of urine sample acquisition, the strong proportional dominance of the detection of Gram-positive rod-shaped bacteria in mid-stream urine samples compared to their low abundance in invasively acquired urine samples makes contamination events in mid-stream urine highly likely. As Gram-positive rod-shaped bacteria are standard colonizers of the human skin, contamination events in the colonized distal urogenital tract may easily occur. The few detections on catheters were restricted to indwelling urinary catheters. For this type of catheter, occlusion due to crystal formation in the course of infections with *Corynebacterium* spp. has been reported [14]. Further, severe *Actinotignum schaalii* catheter-associated infections have been described [51]. The etiological role of a single catheter-associated *A. schaalii*-detection in our study remained, however, unresolved.

In international literature, increased proportions of *Corynebacterium* spp.-infections including urinary tract infections and usually associated with severe, immunocompromising underlying medical conditions were observed in males compared to females [52,53,54]. However, colonization of the urinary tract with *Corynebacterium* spp. was also shown to be common in healthy young males [55]. In the study presented here, only minor differences between male and female patients regarding the detection of Gram-positive rod-shaped bacteria were observed. Focusing on such bacteria with reported potential relevance for urinary tract infections [13,14,15,16,17,18,19,20], *C. aurimucosum* was more frequently observed in females, and *C. glucoronolyticum* and *C. pseudogenitalium* in males. However, such minor differences have to be interpreted with care considering the low total numbers of detections and the uncertain etiological relevance of the respective isolates.

Regarding patient age, increased detection rates of Gram-positive rod-shaped bacteria in patients older than 60 years of age as reported by others [56,57,58] could be shown for *Actinotignum*/*Actinobaculum* spp., while *Corynebacterium* spp. and *Actinomyces*/*Winkia* spp. were evenly distributed over the various age ranges, grouped according to decade. Also, in contrast to reports by others [59,60], *Actinotignum*/*Actinobaculum* spp. isolates were widely missing in young minors. Again, low total numbers might be one reason for the observed discrepancy.

The study has a number of limitations. First, apart from sex and age, no further information on the study population was collected, which is a deviation from the STARD criteria for diagnostic accuracy studies [61] focusing on the assay comparison component of the assessment. In addition, this limitation did not allow any association of detected Gram-positive rod-shaped bacteria with clinical symptoms or clinical courses of the patients. In this respect, the study does not allow any direct conclusions on the likely etiological relevance of the isolated bacteria. Joint study approaches between clinical and laboratory departments might resolve this limitation in future assessments. Of note, however, bacterial species for which potential etiological relevance had previously been suggested [13,14,15,16,17,18,19,20,21,22,23,24,25,26,27,28] were indeed recorded. Second, this interpretative challenge was even aggravated by the fact that the study design neither included quantification of the isolates nor assessments on their occurrence in pure or mixed cultures as well as on the composition of such mixed cultures. Respective sub-stratification would have made interpretations of this hypothesis-forming holistic study approach even more challenging but should be included in confirmatory assessments. Third, detailed information on the storage and transport conditions of the individual samples was not collected, making estimations of the influence of these factors [11] on the likelihood of the detection of Gram-positive rod-shaped bacteria unfeasible. Future studies should consider such interfering factors as well. Fourth, in spite of the high total number of assessed samples, species rarely associated with urinary tract infections were also rarely found in this assessment, making the estimation of the potential relevance of minor quantitative differences challenging. Multicentric-study approaches might resolve this problem in future studies.

## 5. Conclusions

In spite of the abovementioned limitations, the study suggests a number of consequences. The results of the diagnostic standard procedures for the discrimination of Gram-positive rod-shaped bacteria from urinary samples have to be interpreted with care due to imperfect diagnostic accuracy, particularly at the species level. Each laboratory should consider the option of step-wise confirmatory testing, potentially including laborious and time-consuming procedures like sequencing if a species-level identification is considered to be relevant in individual cases. The relevance of age and sex for the isolation of Gram-positive rod-shaped bacteria from urine seems negligible. Detection rates of Gram-positive rod-shaped bacteria are much higher from mid-stream urine compared to invasively sampled urine, stressing the high likelihood of sample contamination in the distal urinary tract. As a consequence, confirmatory testing with invasively sampled urine should be considered if etiological relevance of identified Gram-positive rod-shaped bacteria in urine samples is considered, e.g., in case of repeated detections of such bacteria in subsequently collected urine samples and lacking clinical response to the medical treatment of other likely causative agents of persisting urinary tract infection. In individual cases, prolongated incubation for about 4 days can be considered in the case of suspicion of otherwise undetected urinary tract infections with Gram-positive rod-shaped bacteria, because a minority of the isolates were not recorded after the standard incubation time of 40–48 h.

## Figures and Tables

**Table 1:** Diagnostic flowchart as applied for the study. Applied diagnostic strategies are color-coded in blue, diagnostic results leading to subsequent procedures are shown in green in case of conclusive results as well as in yellow in case of non-conclusive results, and diagnostic results leading to discarding of diagnostic materials are shown in red.

Urine sample for diagnostic urinary culture from patients with clinical suspicion of urinary tract infection					
↓			↓		
2 days of incubation ± subsequent sub-culturing of colonies suspected of containing Gram-positive rod-shaped bacteria			4 days of incubation ± subsequent sub-culturing of colonies suspected of containing Gram-positive rod-shaped bacteria		
↓			↓		
Gram staining					
↓	↓	↓	↓		↓
Gram-negative bacteria	Gram-positive cocci	Gram-positive rod-shaped bacteria	Coccoid Gram-positive bacteria		Non-conclusive result
↓		↓	↓		↓
Discarded		Matrix-assisted laser desorption–ionization time-of-flight mass spectrometry			
		↓	↓		↓
Others			Gram-positive rod-shaped bacteria		Non-conclusive result
↓			↓		↓
Discarded			API (analytical profile index) for coryneform rod-shaped bacteria		
			↓		
16S rRNA gene sequencing					
↓			↓		
Others			Gram-positive rod-shaped bacteria		
↓			↓		
Discarded			Freeze-storage for potential re-testing		

**Table 2:** Characterization of the study population consisting of inpatients with suspicion of urinary tract infection.

Number of Urine Sample Stratified by Age Groups	Male (*n*)	Female (*n*)	Total (*n*)
Total (*n*)	678	492	1170
0–10 years (*n*)	56	33	89
11–20 years (*n*)	43	41	84
21–30 years (*n*)	18	25	43
31–40 years (*n*)	28	31	59
41–50 years (*n*)	69	60	129
51–60 years (*n*)	129	100	229
61–70 years (*n*)	104	57	161
71–80 years (*n*)	182	96	278
81–90 years (*n*)	49	38	87
90+ years (*n*)	0	11	11

*n* = total number.

**Table 3:** Matches and mismatches between the applied analytic approaches.

**Confirmation of mass-spectrometry-based or biochemistry-based results by 16S rRNA gene sequencing**						
	**Confirmation of MALDI-TOF-MS (Saramis database) results by 16S rRNA gene sequencing**		**Confirmation of MALDI-TOF-MS (Myla database) results by 16S rRNA gene sequencing**		**Confirmation of biochemistry (API Coryne) results by 16S rRNA gene sequencing**	
	**At the genus level, *n*/*n* (%)**	**At the species level, *n*/*n* (%)**	**At the genus level, *n*/*n* (%)**	**At the species level, *n*/*n* (%)**	**At the genus level, *n*/*n* (%)**	**At the species level, *n*/*n* (%)**
Gram-positive rod-shaped bacteria	75/115 (65.2%)	25/115 (21.7%)	159/302 (52.6%)	88/302 (29.1%)	212/412 (51.5%)	59/412 (14.3%)
Non-conclusive 16S rRNA gene sequencing results	15/115 (13.0%)		6/302 (2.0%)		0/412 (-) *	
**Matching of mass-spectrometry-based or biochemistry-based results with 16S rRNA gene sequencing if the latter is applied as a reference standard**						
	**Matching of MALDI-TOF-MS (Saramis database) with 16S rRNA gene sequencing**		**Matching of MALDI-TOF-MS (Myla database) with 16S rRNA gene sequencing**		**Matching of biochemistry (API Coryne) with 16S rRNA gene sequencing**	
	**At the genus level, *n*/*n* (%)**	**At the species level, *n*/*n* (%)**	**At the genus level, *n*/*n* (%)**	**At the species level, *n*/*n* (%)**	**At the genus level, *n*/*n* (%)**	**At the species level, *n*/*n* (%)**
*Corynebacterium* spp.	84/254 (33.1%)	15/254 (5.9%)	181/254 (71.3%)	17/254 (6.7%)	172/254 (67.7%)	40/254 (15.7%)
*Actinomyces*/*Winkia* spp.	10/79 (12.7%)	3/79 (3.8%)	60/79 (75.9%)	23/79 (29.1%)	36/79 (45.6%)	18/79 (22.8%)
*Actinobaculum*/*Actinotignum* spp.	6/29 (20.7%)	2/29 (6.9%)	0/29 (-)	0/29 (-)	0/29 (-)	0/29 (-)

* Of note, two API Coryne results were matched by 16S rRNA gene sequencing results on family level only.

**Table 4:** Distribution of the detections of Gram-positive rod-shaped bacteria over the various provided urine sampling approaches, ordered by number.

Distribution of Gram-Positive Rod-Shaped Bacteria over the Different Urine Sampling Approaches			
Urine Sampling Approach	Number (*n*) of Assessed Samples	Number (*n*) of Detections of Gram-Positive Rod-Shaped Bacteria	Proportion (%) of Samples with Growth of Gram-Positive Rod-Shaped Bacteria
Mid-stream urine	795	358	45.0%
Urine from an Indwelling urinary catheter	147	4	2.7%
Unknown urine sampling approach	102	38	37.3%
Urine collected in a pouch	38	7	18.4%
Urine from an intermittent urinary catheter	20	0	-
Urine from a urinary catheter without further information	18	0	-
Urine from an entero-vesical fistula	18	1	5.6%
Urine from an ileal conduit	13	1	7.7%
Urine collected in a pot	10	1	10.0%
Urine collected via bladder puncture	6	0	-
Urine collected via puncture of the renal pelvis	2	0	-
First jet urine	1	1	100.0%
**Distribution of *Corynebacterium* spp., *Actinomyces*/*Winkia* spp. and *Actinotignum*/*Actinobaculum* spp. over Major Clusters of Different Urine Sampling Approaches**			
**Genus**	**Mid-Stream Urine**	**Urine from a Catheter**	**Other Types of Urine Sampling**
*Corynebacterium*	223 (54.1%)	1 (0.2%)	30 (7.3%)
*Actinomyces*/*Winkia*	65 (15.8%)	1 (0.2%)	13 (3.2%)
*Actinotignum/Actinobaculum*	25 (6.1%)	1 (0.2%)	3 (0.7%)

**Table 5:** Distribution of *Corynebacterium* spp., *Actinomyces*/*Winkia* spp. and *Actinotignum*/*Actinobaculum* spp. by female and male sex.

Genus	Male (*n*)	Female (*n*)
*Corynebacterium*	60	64
*Actinomyces*/*Winkia*	22	16
*Actinotignum/Actinobaculum*	8	7

**Table 6:** Distribution of Gram-positive rod-shaped bacteria over the various age in decades of the assessed patients.

	Age in Decades									
	0–10	11–20	21–30	31–40	41–50	51–60	61–70	71–80	81–90	90+
Number of urine samples	89	84	43	59	129	229	161	278	87	11
Proportion of 1170 urine samples	7.6	7.2	3.7	5.0	11.0	19.6	13.8	23.8	7.4	0.9
Number of Gram-positive rod-shaped bacteria	24	31	16	26	55	98	51	86	21	4
Average number needed to detect a Gram-positive rod-shaped bacterium	3.7	2.7	2.7	2.3	2.3	2.3	3.2	3.2	4.1	2.8

**Table 7:** Distribution of *Corynebacterium* spp., *Actinomyces*/*Winkia* spp. and *Actinotignum*/*Actinobaculum* spp. by age decade of the assessed patients.

Age Decade in Years	0–10	11–20	21–30	31–40	41–50	51–60	61–70	71–80	81–90	>90
Number of urine assessments	89	84	43	59	129	229	161	278	87	11
Number of *Corynebacterium* spp. detections	15	20	14	12	35	67	30	48	13	2
Number of *Corynebacterium* spp. detections per urine assessment	0.17	0.24	0.33	0.20	0.27	0.29	0.19	0.17	0.15	0.18
Number of *Actinomyces*/*Winkia* spp. detections	7	7	1	8	12	16	10	18	1	1
Number of *Actinomyces*/*Winkia* spp. detections per urine assessment	0.08	0.08	0.02	0.14	0.09	0.07	0.06	0.06	0.01	0.09
Number of *Actinotignum/Actinobaculum* spp. detections	1	0	0	2	2	5	7	8	3	1
Number of *Actinotignum/Actinobaculum* spp. detections per urine assessment	0.01	0.00	0.00	0.03	0.02	0.02	0.04	0.03	0.03	0.09

## Data Availability

All relevant data are provided in the manuscript and Appendix A. Raw data can be made available upon reasonable request. Sequence data are not deposited in databases, because sequencing has been used for diagnostic purposes, and so the identity of the assessed microorganisms cannot be considered as definitely guaranteed.

## Appendix A

**Table A1:** Diagnostic results as obtained with the different applied identification approaches in alphabetic order. Results are presented exactly as provided by the applied databases, explaining the partly outdated nomenclature.

Diagnostic Result	MALDI-TOF-MS (Saramis Database, *n* = 441), *n* (%)	MALDI-TOF-MS (Myla Database, *n* = 441), *n* (%)	Biochemical Differentiation (API Coryne, *n* = 452), *n* (%)	16S rRNA Gene Sequencing (*n* = 412), *n* (%)
*Actinobaculum massiliense*	-	-	-	1 (0.2%)
*Actinobaculum schaalii*	11 (2.5%)	-	-	18 (4.4%)
*Actinobaculum* sp. V04 257809/08	-	-	-	10 (2.4%)
*Actinomyces europaeus*	-	1 (0.2%)		4 (1.0%)
*Actinomyces hominis*	-	-	-	1 (0.2%)
*Actinomyces neuii*	2 (0.5%)	29 (7.4%)	-	4 (1.0%)
*Actinomyces neuii* ssp. *anitratus*	-	-	17 (3.8%)	-
*Actinomyces neuii* ssp. *neuii*	-	-	12 (2.7%)	-
*Actinomyces radingae*	-	10 (2.5%)	12 (2.7%)	6 (1.5%)
*Actinomyces radingae/Aerococcus viridans*	-	1 (0.2%)	-	-
*Actinomyces* sp.	7 (1.6%)	-	-	-
*Actinomyces* sp. 13-114	-	-	-	1 (0.2%)
*Actinomyces* sp. 13-605	-	-	-	8 (1.9%)
*Actinomyces* sp. 2234/04	-	-	-	2 (0.5%)
*Actinomyces* sp. “ARUP UnID60”	-	-	-	26 (6.3%)
*Actinomyces* sp. S5-BM9	-	-	-	1 (0.2%)
*Actinomyces* sp. SD1	-	-	-	5 (1.2%)
*Actinomyces turicensis*	2 (0.5%)	38 (9.7%)	41 (9.1%)	17 (4.1%)
*Actinomyces urogenitalis*	-	-	-	4 (1.0%)
*Alloscardovia omnicolens*	-	-	-	9 (2.2%)
*Arcanobacterium pyogenes*	-	-	2 (0.4%)	-
*Arcanobacterium pyogenes/Brevibacterium* spp.	-	-	1 (0.2%)	-
*Arthrobacter albus*	-	-	-	1 (0.2%)
*Arthrobacter cumminsii*	-	2 (0.5%)	-	1 (0.2%)
*Arthrobacter* spp.	-	-	23 (5.1%)	-
*Bacillus badius*	-	3 (0.8%)		-
*Bacillus* sp. PD1B	-	-	-	1 (0.2%)
*Bacillus thuringiensis*	-	1 (0.2%)	-	-
bacterium str. Rauti	-	-	-	3 (0.7%)
*Brevibacterium* spp.	-	-	30 (6.6%)	-
*Brevibacterium* spp./*Arcanobacterium bernardiae*/*Gardnerella vaginalis*	-	-	1 (0.2%)	-
*Brevibacterium* spp./*Gardnerella vaginalis*/*Actinomyces neuii* ssp. *anitratus*	-	-	1 (0.2%)	-
*Cellumonas* spp./*Microbacterium* spp.	-	-	7 (1.5%)	-
*Clostridium cadaveris*	-	1 (0.2%)	-	-
*Clostridium clostridioforme*	-	1 (0.2%)	-	-
*Clostridium histolyticum*	-	1 (0.2%)	-	-
*Corynebacteriaceae bacterium* “ARUP UnID261”	-	-	-	1 (0.2%)
*Corynebacteriaceae bacterium* “ARUP UnID268”	-	-	-	1 (0.2%)
*Corynebacterium afermentans*	-	-	-	1 (0.2%)
*Corynebacterium afermentans/coyleae*	-	-	14 (3.1%)	-
*Corynebacterium amycolatum*	1 (0.2%)	-	-	7 (1.7%)
*Corynebacterium amycolatum/jeikeium/xerosis*	1 (0.2%)	-	-	-
*Corynebacterium amycolatum/xerosis*	51 (11.6%)	85 (21.7%)	1 (0.2%)	-
*Corynebacterium argentoratense*	-	-	19 (4.2%)	-
*Corynebacterium aurimucosum*	-	7 (1.8%)	-	11 (2.7%)
*Corynebacterium aurimucosum/Rhodococcus erythropolis*	-	1 (0.2%)	-	-
*Corynebacterium auris/Turicella otitidis*	-	-	15 (3.3%)	-
*Corynebacterium bovis*	-	-	6 (1.3%)	-
*Corynebacterium confusum*	-	1 (0.2%)	-	1 (0.2%)
*Corynebacterium coyleae*	-	11 (2.8%)	-	2 (0.5%)
*Corynebacterium freneyi*	-	2 (0.5%)	-	-
*Corynebacterium glucuronolyticum*	18 (4.1%)	50 (12.8%)	55 (12.2%)	42 (10.2%)
*Corynebacterium glucuronolyticum/Arcanobacterium pyogenes/Gardnerella vaginalis/Arcanobacterium haemolyticum*	-	-	1 (0.2%)	-
*Corynebacterium* group F1	-	-	5 (1.1%)	-
*Corynebacterium* group G	-	-	6 (1.3%)	-
*Corynebacterium imitans*	-	-	-	4 (1.0%)
*Corynebacterium jeikeium*	1 (0.2%)	5 (1.3%)	22 (4.9%)	3 (0.7%)
*Corynebacterium macginleyi*	1 (0.2%)	1 (0.2%)	2 (0.4%)	1 (0.2%)
*Corynebacterium massiliense*	-	-	-	1 (0.2%)
*Corynebacterium minutissimum*	-	-	-	3 (0.7%)
*Corynebacterium mucifaciens*	-	1 (0.2%)	-	-
*Corynebacterium propinquum*	-	-	66 (14.6%)	-
*Corynebacterium pseudodiphtheriticum*	-	-	4 (0.9%)	-
*Corynebacterium pseudogenitalium*	-	-	-	29 (7.0%)
*Corynebacterium pseudotuberculosis*	-	-	5 (1.1%)	-
*Corynebacterium renale*	-	1 (0.2%)	-	-
*Corynebacterium riegelii*	-	-	-	1 (0.2%)
*Corynebacterium simulans*	-	-	-	1 (0.2%)
*Corynebacterium singulare*	-	-	-	1 (0.2%)
*Corynebacterium* sp.	18 (4.1%)	-	-	9 (2.2%)
*Corynebacterium* sp. 2012257588	-	-	-	1 (0.2%)
*Corynebacterium* sp. 2012259355	-	-	-	1 (0.2%)
*Corynebacterium* sp. 31595	-	-	-	4 (1.0%)
*Corynebacterium* sp. 59614	-	-	-	1 (0.2%)
*Corynebacterium* sp. 707471/2012	-	-	-	12 (2.9%)
*Corynebacterium* sp. “ARUP UnID231”	-	-	-	1 (0.2%)
*Corynebacterium* sp. “ARUP UnID245”	-	-	-	3 (0.7%)
*Corynebacterium* sp. “ARUP UnID281”	-	-	-	5 (1.2%)
*Corynebacterium* sp. “ARUP UnID60”	-	-	-	1 (0.2%)
*Corynebacterium* sp. ATCC 6931	-	-	-	9 (2.2%)
*Corynebacterium* sp. canine oral taxon 424, OH 977	-	-	-	4 (1.0%)
*Corynebacterium* sp. M3T9B3	-	-	-	2 (0.5%)
*Corynebacterium* sp. MOLA 35	-	-	-	1 (0.2%)
*Corynebacterium* sp. NML96-0085	-	-	-	5 (1.2%)
*Corynebacterium* sp. NML90-0020	-	-	-	1 (0.2%)
*Corynebacterium* sp. S504	-	-	-	1 (0.2%)
*Corynebacterium* sp. “Smarlab Biomol”	-	-	-	67 (16.3%)
*Corynebacterium* sp. R-45865	-	-	-	5 (1.2%)
*Corynebacterium* sp. R603	-	-	-	2 (0.5%)
*Corynebacterium striatum*	-	1 (0.2%)	-	-
*Corynebacterium striatum/amycolatum*	-	-	33 (7.3%)	-
*Corynebacterium tuberculostearicum*	-	33 (8.4%)	-	5 (1.2%)
*Corynebacterium urealyticum*	1 (0.2%)	-	9 (2.0%)	2 (0.5%)
*Corynebacterium ureicelerivorans*	-	-	-	2 (0.5%)
*Corynebacterium xerosis*	-	1 (0.2%)	-	2 (0.5%)
*Dermabacter hominis*	1 (0.2%)	2 (0.5%)	6 (1.3%)	2 (0.5%)
*Dermabacter* sp. AD186	-	-	-	1 (0.2%)
*Erysipelothrix rhusiopathiae*	-	-	4 (0.9%)	-
*Gardnerella vaginalis*	-	-	15 (3.3%)	-
*Gardnerella vaginalis/Erysipelothrix rhusiopathiae/Brevibacterium* spp./*Listeria monocytogenes*/*innocua*/*Propionibacterium avidum*	-	-	1 (0.2%)	-
*Gardnerella vaginalis/Propionibacterium avidum*	-	-	1 (0.2%)	-
*Listeria weishimeri*	-	1 (0.2%)	-	-
*Lysinibacillus sphaericus*	-	1 (0.2%)	-	-
*Microbacterium arborescens*	-	1 (0.2%)	-	-
*Mycobacterium bovis/fortuitum/tuberculosis*	-	1 (0.2%)	-	-
*Mycobakterium intracellulare*	-	3 (0.8%)	-	-
*Mycobacterium kansasii*	-	1 (0.2%)	-	-
*Mycobacterium smegmatis*	-	1 (0.2%)	-	-
No identification result	326 (73.9%)	89 (22.8%)	1 (0.2%)	21 (5.1%)
*Paenibacillus durus*	-	1 (0.2%)	-	-
*Propionibacterium acnes*	-	-	2 (0.4%)	-
*Propionibacterium acnes/Arthrobacter* spp./*Actinomyces radingae*	-	-	2 (0.4%)	-
*Propionibacterium avidum*	-	2 (0.5%)	2 (0.4%)	-
*Pseudoclavibacter bifida*	-	-	-	1 (0.2%)
*Pseudoclavibacter faecalis*	-	-	-	1 (0.2%)
*Rhodococcus* sp.	-	-	7 (1.5%)	-
*Rothia dentocariosa*	-	-	1 (0.2%)	-
uncultured bacterium clone JSC7-39	-	-	-	1 (0.2%)
*Zimmermannella* sp. “ARUP UnID673”	-	-	-	1 (0.2%)

**Table A2:** Proportions (in %) of agar plates of 1170 assessed urine samples focusing on the detection of Gram-positive rod-shaped bacteria comparing day 2 and day 4 of growth.

	Growth of Gram-Positive Rod-Shaped Bacteria on Day 2	No growth of Gram-Positive Rod-Shaped Bacteria on Day 2
Additional growth of Gram-positive rod-shaped bacteria on day 4	11%	4%
No additional growth of Gram-positive rod-shaped bacteria on day 4	59%	26%

**Table A3:** Microorganisms isolated from agar plates showing no growth of Gram-positive rod-shaped bacteria at day 2 but from which Gram-positive rod-shaped bacteria were isolated at day 4. Ordered by number of isolation events. Results are presented exactly as provided by the applied database, explaining the partly outdated nomenclature.

Species	Number (*n*)
*Actinobaculum schaalii*	3
*Actinomyces* sp. “ARUP UnID60”	3
*Corynebacterium glucuronolyticum*	3
*Corynebacterium* sp. 707471/2012	3
*Alloscardovia omnicolens*	2
*Corynebacterium pseudogenitalium*	2
Not identified	2
*Actinobaculum* sp. V04 257809/08	1
*Actinomyces turicensis*	1
bacterium str. Rauti	1
*Corynebacterium imitans*	1
*Corynebacterium* sp.	1
*Corynebacterium* sp. “Smarlab Biomol”	1
*Corynebacterium* sp. R-45865	1
*Corynebacterium tuberculostearicum*	1

**Table A4:** Microorganisms isolated from agar plates showing growth of Gram-positive rod-shaped bacteria at day 2 and from which additional Gram-positive rod-shaped bacteria were isolated at day 4. Ordered by number of isolation events. Results are presented exactly as provided by the applied database, explaining the partly outdated nomenclature.

Species	Number (*n*)
*Corynebacterium* sp. “Smarlab Biomol”	15
*Corynebacterium glucuronolyticum*	13
*Actinomyces turicensis*	6
*Corynebacterium pseudogenitalium*	6
*Actinomyces* sp. “ARUP UnID60”	5
Not identified	4
*Actinomyces* sp. 13-605	3
*Corynebacterium amycolatum*	3
*Corynebacterium* sp. 707471/2012	3
*Corynebacterium* sp. R-45865	3
*Actinobaculum schaalii*	2
*Actinobaculum* sp. V04 257809/08	2
*Actinomyces* sp. SD1	2
*Corynebacterium* sp.	2
*Corynebacterium* sp. 31595	2
*Actinomyces europaeus*	1
*Actinomyces* sp. 2234/04	1
*Actinomyces urogenitalis*	1
*Alloscardovia omnicolens*	1
*Bacillus* sp. PD1B	1
*Brevibacterium* sp. TSW19BA7	1
*Corynebacterium afermentans*	1
*Corynebacterium aurimucosum*	1
*Corynebacterium massiliense*	1
*Corynebacterium singulare*	1
*Corynebacterium* sp. “ARUP UnID245”	1
*Corynebacterium* sp. “ARUP UnID60”	1
*Corynebacterium* sp. ATCC 6931	1
*Corynebacterium* sp. NML90-0020	1
*Corynebacterium* sp. NML96-0085	1
*Corynebacterium* sp. R603	1
*Corynebacterium urealyticum*	1
*Pseudoclavibacter bifida*	1
*Pseudoclavibacter faecalis*	1

**Table A5:** Distribution of the species as assessed with 16S rRNA gene sequencing over the different urine sampling approaches, ordered by number. Results are presented exactly as provided by the applied database, explaining the partly outdated nomenclature.

Species	Total	Mid-Stream Urine	Unknown Urine Sampling Approach	Urine Sampled in a Pouch	Urine from an Indwelling Catheter	Urine from an Ileal Conduit	Urine from an Intermittent Urinary Catheter	Urine from an Entero-Vesical Fistula	Urine Collected in a Pot
*Corynebacterium* sp. “Smarlab Biomol”	67	59	6	0	1	0	0	1	0
*Corynebacterium glucuronolyticum*	42	39	3	0	0	0	0	0	0
*Corynebacterium pseudogenitalium*	29	25	1	2	0	1	0	0	0
*Actinomyces* sp. “ARUP UnID60”	26	22	2	1	0	0	1	0	0
Not identified	22	18	4	0	0	0	0	0	0
*Actinobaculum schaalii*	18	16	1	0	1	0	0	0	0
*Actinomyces turicensis*	17	12	1	3	1	0	0	0	0
*Corynebacterium* sp. 707471/2012	12	11	0	0	0	0	1	0	0
*Corynebacterium aurimucosum*	11	8	3	0	0	0	0	0	0
*Actinobaculum* sp. V04 257809/08	10	8	2	0	0	0	0	0	0
*Alloscardovia omnicolens*	9	7	2	0	0	0	0	0	0
*Corynebacterium* sp.	9	9	0	0	0	0	0	0	0
*Corynebacterium* sp. ATCC 6931	9	9	0	0	0	0	0	0	0
*Actinomyces* sp. 13-605	8	6	2	0	0	0	0	0	0
*Corynebacterium amycolatum*	7	6	1	0	0	0	0	0	0
*Actinomyces radingae*	6	5	1	0	0	0	0	0	0
*Actinomyces* sp. SD1	5	5	0	0	0	0	0	0	0
*Corynebacterium* sp. “ARUP UnID281”	5	5	0	0	0	0	0	0	0
*Corynebacterium* sp. NML96-0085	5	5	0	0	0	0	0	0	0
*Corynebacterium* sp. R-45865	5	4	1	0	0	0	0	0	0
*Corynebacterium tuberculostearicum*	5	4	1	0	0	0	0	0	0
*Actinomyces europaeus*	4	4	0	0	0	0	0	0	0
*Actinomyces neuii*	4	4	0	0	0	0	0	0	0
*Actinomyces urogenitalis*	4	2	1	1	0	0	0	0	0
*Corynebacterium imitans*	4	3	1	0	0	0	0	0	0
*Corynebacterium* sp. 31595	4	3	0	0	0	0	0	0	1
*Corynebacterium* sp. canine oral taxon 424, OH 977	4	4	0	0	0	0	0	0	0
bacterium str. Rauti	3	3	0	0	0	0	0	0	0
*Corynebacterium jeikeium*	3	3	0	0	0	0	0	0	0
*Corynebacterium minutissimum*	3	3	0	0	0	0	0	0	0
*Corynebacterium* sp. “ARUP UnID245”	3	2	1	0	0	0	0	0	0
*Actinomyces* sp. 2234/04	2	2	0	0	0	0	0	0	0
*Brevibacterium paucivorans*	2	0	1	0	1	0	0	0	0
*Brevibacterium* sp. TSW19BA7	2	2	0	0	0	0	0	0	0
*Corynebacterium coyleae*	2	2	0	0	0	0	0	0	0
*Corynebacterium* sp. M3T9B3	2	2	0	0	0	0	0	0	0
*Corynebacterium* sp. R603	2	2	0	0	0	0	0	0	0
*Corynebacterium urealyticum*	2	1	1	0	0	0	0	0	0
*Corynebacterium ureicelerivorans*	2	2	0	0	0	0	0	0	0
*Corynebacterium xerosis*	2	1	1	0	0	0	0	0	0
*Dermabacter hominis*	2	2	0	0	0	0	0	0	0
*Actinobaculum massiliense*	1	1	0	0	0	0	0	0	0
*Actinomyces hominis*	1	1	0	0	0	0	0	0	0
*Actinomyces* sp. 13-114	1	1	0	0	0	0	0	0	0
*Actinomyces* sp. S5-BM9	1	1	0	0	0	0	0	0	0
*Arthrobacter albus*	1	1	0	0	0	0	0	0	0
*Arthrobacter cumminsii*	1	1	0	0	0	0	0	0	0
*Bacillus* sp. PD1B	1	1	0	0	0	0	0	0	0
*Corynebacteriaceae bacterium* “ARUP UnID261”	1	1	0	0	0	0	0	0	0
*Corynebacteriaceae bacterium* “ARUP UnID268”	1	1	0	0	0	0	0	0	0
*Corynebacterium afermentans*	1	1	0	0	0	0	0	0	0
*Corynebacterium confusum*	1	1	0	0	0	0	0	0	0
*Corynebacterium macginleyi*	1	1	0	0	0	0	0	0	0
*Corynebacterium massiliense*	1	1	0	0	0	0	0	0	0
*Corynebacterium riegelii*	1	1	0	0	0	0	0	0	0
*Corynebacterium simulans*	1	1	0	0	0	0	0	0	0
*Corynebacterium singulare*	1	1	0	0	0	0	0	0	0
*Corynebacterium* sp. “ARUP UnID231”	1	1	0	0	0	0	0	0	0
*Corynebacterium* sp. “ARUP UnID60”	1	1	0	0	0	0	0	0	0
*Corynebacterium* sp. 2012257588	1	1	0	0	0	0	0	0	0
*Corynebacterium* sp. 2012259355	1	0	1	0	0	0	0	0	0
*Corynebacterium* sp. 59614	1	1	0	0	0	0	0	0	0
*Corynebacterium* sp. MOLA 35	1	1	0	0	0	0	0	0	0
*Corynebacterium* sp. NML90-0020	1	1	0	0	0	0	0	0	0
*Corynebacterium* sp. S504	1	1	0	0	0	0	0	0	0
*Dermabacter* sp. AD186	1	1	0	0	0	0	0	0	0
*Pseudoclavibacter bifida*	1	1	0	0	0	0	0	0	0
*Pseudoclavibacter faecalis*	1	1	0	0	0	0	0	0	0
uncultured bacterium clone JSC7-39	1	1	0	0	0	0	0	0	0
*Zimmermannella* sp. “ARUP UnID673”	1	1	0	0	0	0	0	0	0

**Table A6:** Distribution of the of the species as assessed with 16S rRNA gene sequencing over the female and male sex, ordered by number. Results are presented exactly as provided by the applied database, explaining the partly outdated nomenclature.

Species	Total	Male	Female
*Corynebacterium* sp. “Smarlab Biomol”	67	11	56
*Corynebacterium glucuronolyticum*	42	38	4
*Corynebacterium pseudogenitalium*	29	24	5
*Actinomyces* sp. “ARUP UnID60”	26	15	11
Not identified	22	11	11
*Actinobaculum schaalii*	18	12	6
*Actinomyces turicensis*	17	13	4
*Corynebacterium* sp. 707471/2012	12	12	0
*Corynebacterium aurimucosum*	11	3	8
*Actinobaculum* sp. V04 257809/08	10	5	5
*Alloscardovia omnicolens*	9	4	5
*Corynebacterium* sp.	9	9	0
*Corynebacterium* sp. ATCC 6931	9	1	8
*Actinomyces* sp. 13-605	8	5	3
*Corynebacterium amycolatum*	7	1	6
*Actinomyces radingae*	6	6	0
*Actinomyces* sp. SD1	5	5	0
*Corynebacterium* sp. “ARUP UnID281”	5	1	4
*Corynebacterium* sp. NML96-0085	5	2	3
*Corynebacterium* sp. R-45865	5	5	0
*Corynebacterium tuberculostearicum*	5	4	1
*Actinomyces europaeus*	4	2	2
*Actinomyces neuii*	4	2	2
*Actinomyces urogenitalis*	4	2	2
*Corynebacterium imitans*	4	2	2
*Corynebacterium* sp. 31595	4	3	1
*Corynebacterium* sp. canine oral taxon 424, OH 977	4	3	1
bacterium str. Rauti	3	2	1
*Corynebacterium jeikeium*	3	3	0
*Corynebacterium minutissimum*	3	2	1
*Corynebacterium* sp. “ARUP UnID245”	3	0	3
*Actinomyces* sp. 2234/04	2	0	2
*Brevibacterium paucivorans*	2	2	0
*Brevibacterium* sp. TSW19BA7	2	0	2
*Corynebacterium coyleae*	2	1	1
*Corynebacterium* sp. M3T9B3	2	0	2
*Corynebacterium* sp. R603	2	1	1
*Corynebacterium urealyticum*	2	0	2
*Corynebacterium ureicelerivorans*	2	1	1
*Corynebacterium xerosis*	2	2	0
*Dermabacter hominis*	2	2	0
*Actinobaculum massiliense*	1	0	1
*Actinomyces hominis*	1	0	1
*Actinomyces* sp. 13-114	1	0	1
*Actinomyces* sp. S5-BM9	1	0	1
*Arthrobacter albus*	1	0	1
*Arthrobacter cumminsii*	1	0	1
*Bacillus* sp. PD1B	1	1	0
*Corynebacteriaceae bacterium* “ARUP UnID261”	1	0	1
*Corynebacteriaceae bacterium* “ARUP UnID268”	1	1	0
*Corynebacterium afermentans*	1	1	0
*Corynebacterium confusum*	1	0	1
*Corynebacterium macginleyi*	1	0	1
*Corynebacterium massiliense*	1	0	1
*Corynebacterium riegelii*	1	0	1
*Corynebacterium simulans*	1	0	1
*Corynebacterium singulare*	1	1	0
*Corynebacterium* sp. “ARUP UnID231”	1	1	0
*Corynebacterium* sp. “ARUP UnID60”	1	1	0
*Corynebacterium* sp. 2012257588	1	1	0
*Corynebacterium* sp. 2012259355	1	1	0
*Corynebacterium* sp. 59614	1	0	1
*Corynebacterium* sp. MOLA 35	1	0	1
*Corynebacterium* sp. NML90-0020	1	1	0
*Corynebacterium* sp. S504	1	0	1
*Dermabacter* sp. AD186	1	1	0
*Pseudoclavibacter bifida*	1	0	1
*Pseudoclavibacter faecalis*	1	1	0
uncultured bacterium clone JSC7-39	1	0	1
*Zimmermannella* sp. “ARUP UnID673”	1	0	1

**Table A7:** Distribution of the of the species as assessed with 16S rRNA gene sequencing over the age in decades of the assessed patients, ordered by number. Results are presented exactly as provided by the applied database, explaining the partly outdated nomenclature.

Species	Age in Decades										
	0–10	11–20	21–30	31–40	41–50	51–60	61–70	71–80	81–90	90+	Total
*Corynebacterium* sp. “Smarlab Biomol”	4	5	2	2	12	16	8	14	4	0	67
*Corynebacterium glucuronolyticum*	3	3	3	2	5	10	7	9	0	0	42
*Corynebacterium pseudogenitalium*	4	3	2	1	3	4	2	7	3	0	29
*Actinomyces* sp. “ARUP UnID60”	1	2	0	2	6	7	5	3	0	0	26
Not identified	0	1	0	3	1	8	1	5	3	0	22
*Actinotignum schaalii*	1	0	0	2	1	4	3	3	3	1	18
*Actinomyces turicensis*	3	2	1	4	0	1	2	4	0	0	17
*Corynebacterium* sp. 707471/2012	0	1	0	0	1	4	1	3	2	0	12
*Corynebacterium aurimucosum*	0	0	0	1	1	4	1	3	1	0	11
*Actinobaculum* sp. V04 257809/08	0	0	0	0	1	0	4	5	0	0	10
*Alloscardovia omnicolens*	0	1	1	1	2	2	0	2	0	0	9
*Corynebacterium* sp.	0	1	1	1	2	1	2	0	1	0	9
*Corynebacterium* sp. ATCC 6931	0	0	2	0	0	5	1	1	0	0	9
*Actinomyces* sp. 13-605	0	0	0	1	1	5	0	1	0	0	8
*Corynebacterium amycolatum*	0	1	0	0	1	1	0	3	1	0	7
*Actinomyces radingae*	0	0	0	0	2	0	1	3	0	0	6
*Actinomyces* sp. SD1	0	3	0	0	0	1	1	0	0	0	5
*Corynebacterium* sp. “ARUP UnID281”	0	1	0	0	2	2	0	0	0	0	5
*Corynebacterium* sp. NML96-0085	0	1	0	0	0	2	1	1	0	0	5
*Corynebacterium* sp. R-45865	0	0	0	1	1	1	1	1	0	0	5
*Corynebacterium tuberculostearicum*	0	1	1	0	0	0	1	2	0	0	5
*Actinomyces europaeus*	0	0	0	0	0	0	1	2	0	1	4
*Actinomyces neuii* LCDC	0	0	0	0	0	1	0	2	1	0	4
*Actinomyces urogenitalis*	1	0	0	1	2	0	0	0	0	0	4
*Corynebacterium imitans*	0	0	2	0	1	0	0	0	0	1	4
*Corynebacterium* sp. 31595	2	0	1	0	0	0	0	0	0	1	4
*Corynebacterium* sp. canine oral taxon 424, OH 977	0	0	0	0	2	2	0	0	0	0	4
bacterium str. Rauti	1	0	0	0	1	0	1	0	0	0	3
*Corynebacterium jeikeium*	0	0	0	1	1	0	0	1	0	0	3
*Corynebacterium minutissimum*	0	1	0	0	0	2	0	0	0	0	3
*Corynebacterium* sp. “ARUP UnID245”	0	0	0	0	0	0	2	1	0	0	3
*Actinomyces* sp. 2234/04	1	0	0	0	0	0	0	1	0	0	2
*Brevibacterium paucivorans*	0	0	0	0	0	0	0	2	0	0	2
*Brevibacterium* sp. TSW19BA7	0	1	0	0	0	0	0	1	0	0	2
*Corynebacterium coyleae*	0	0	0	0	0	2	0	0	0	0	2
*Corynebacterium* sp. M3T9B3	0	1	0	0	0	1	0	0	0	0	2
*Corynebacterium* sp. R603	0	0	0	1	1	0	0	0	0	0	2
*Corynebacterium urealyticum*	0	0	0	0	0	1	0	1	0	0	2
*Corynebacterium ureicelerivorans*	0	0	0	0	0	1	1	0	0	0	2
*Corynebacterium xerosis*	1	0	0	1	0	0	0	0	0	0	2
*Dermabacter hominis*	0	0	0	0	1	0	1	0	0	0	2
*Actinobaculum massiliense*	0	0	0	0	0	1	0	0	0	0	1
*Actinomyces hominis*	0	0	0	0	0	0	0	1	0	0	1
*Actinomyces* sp. 13-114	1	0	0	0	0	0	0	0	0	0	1
*Actinomyces* sp. S5-BM9	0	0	0	0	0	0	0	1	0	0	1
*Arthrobacter albus*	0	0	0	0	1	0	0	0	0	0	1
*Arthrobacter cumminsii*	0	0	0	0	0	1	0	0	0	0	1
*Bacillus* sp. PD1B	0	0	0	0	0	0	1	0	0	0	1
*Corynebacteriaceae bacterium* “ARUP UnID261”	0	0	0	0	0	0	0	1	0	0	1
*Corynebacteriaceae bacterium* “ARUP UnID268”	0	0	0	0	0	0	1	0	0	0	1
*Corynebacterium afermentans*	0	0	0	0	0	1	0	0	0	0	1
*Corynebacterium confusum*	0	0	0	0	0	0	0	0	1	0	1
*Corynebacterium macginleyi*	0	0	0	0	0	1	0	0	0	0	1
*Corynebacterium massiliense*	0	0	0	0	0	0	1	0	0	0	1
*Corynebacterium riegelii*	0	0	0	0	0	1	0	0	0	0	1
*Corynebacterium simulans*	0	0	0	0	0	1	0	0	0	0	1
*Corynebacterium singulare*	0	0	0	0	0	1	0	0	0	0	1
*Corynebacterium* sp. “ARUP UnID231”	0	1	0	0	0	0	0	0	0	0	1
*Corynebacterium* sp. “ARUP UnID60”	0	0	0	0	0	1	0	0	0	0	1
*Corynebacterium* sp. 2012257588	1	0	0	0	0	0	0	0	0	0	1
*Corynebacterium* sp. 2012259355	0	0	0	0	1	0	0	0	0	0	1
*Corynebacterium* sp. 59614	0	0	0	0	1	0	0	0	0	0	1
*Corynebacterium* sp. MOLA 35	0	0	0	0	0	1	0	0	0	0	1
*Corynebacterium* sp. NML90-0020	0	0	0	1	0	0	0	0	0	0	1
*Corynebacterium* sp. S504	0	0	0	0	0	1	0	0	0	0	1
*Dermabacter* sp. AD186	0	0	0	0	0	0	0	1	0	0	1
*Pseudoclavibacter bifida*	0	0	0	0	1	0	0	0	0	0	1
*Pseudoclavibacter faecalis*	0	0	0	0	0	0	0	0	1	0	1
uncultured bacterium clone JSC7-39	0	1	0	0	0	0	0	0	0	0	1
*Zimmermannella* sp. “ARUP UnID673”	0	0	0	0	0	0	0	1	0	0	1
